# Introduction of AI Technology for Objective Physical Function Assessment

**DOI:** 10.3390/bioengineering11111154

**Published:** 2024-11-16

**Authors:** Nobuji Kouno, Satoshi Takahashi, Masaaki Komatsu, Yusuke Sakaguchi, Naoaki Ishiguro, Katsuji Takeda, Kyoko Fujioka, Ayumu Matsuoka, Maiko Fujimori, Ryuji Hamamoto

**Affiliations:** 1 Division of Medical AI Research and Development, National Cancer Center Research Institute, 5-1-1 Tsukiji, Chuo-ku, Tokyo 104-0045, Japan; satoshi.takahashi.fy@riken.jp (S.T.); maskomat@ncc.go.jp (M.K.); yusakag2@ncc.go.jp (Y.S.); kfujioka@ncc.go.jp (K.F.); 2 Cancer Translational Research Team, RIKEN Center for Advanced Intelligence Project, 1-4-1 Nihonbashi, Chuo-ku, Tokyo 103-0027, Japan; naoaki.ishiguro@riken.jp (N.I.); katsuji.takeda@riken.jp (K.T.); 3 Department of Surgery, Graduate School of Medicine, Kyoto University, Yoshida-konoe-cho, Sakyo-ku, Kyoto 606-8507, Japan; 4 Department of Neurosurgery, Faculty of Medicine, The University of Tokyo, 7-3-1 Hongo, Bunkyo-ku, Tokyo 113-8655, Japan; 5 Division of Survivorship Research, National Cancer Center Institute for Cancer Control, 5-1-1 Tsukiji, Chuo-ku, Tokyo 104-0045, Japan; aymatsuo@ncc.go.jp (A.M.); mfujimor@ncc.go.jp (M.F.)

**Keywords:** objective physical function assessment, short physical performance battery, timed up and go, walking speed, grip strength, machine learning

## Abstract

Objective physical function assessment is crucial for determining patient eligibility for treatment and adjusting the treatment intensity. Existing assessments, such as performance status, are not well standardized, despite their frequent use in daily clinical practice. This paper explored how artificial intelligence (AI) could predict physical function scores from various patient data sources and reviewed methods to measure objective physical function using this technology. This review included relevant articles published in English that were retrieved from PubMed. These studies utilized AI technology to predict physical function indices from patient data extracted from videos, sensors, or electronic health records, thereby eliminating manual measurements. Studies that used AI technology solely to automate traditional evaluations were excluded. These technologies are recommended for future clinical systems that perform repeated objective physical function assessments in all patients without requiring extra time, personnel, or resources. This enables the detection of minimal changes in a patient’s condition, enabling early intervention and enhanced outcomes.

## 1. Introduction

Physical function assessment is crucial for clinical decision-making and for guiding treatment strategies across various medical specialties. Surgical treatment, as well as medical treatments, such as dialysis and respiratory management, have an invasive aspect, and physical function is an extremely important indicator determining whether the patient can overcome such invasiveness and accept the therapeutic benefits [1]. Medical professionals use one or more physical function assessment tools, medical history, and hospital impressions to determine treatment strategies. Even among patients of the same age and disease status, variations in physical functions can lead to different treatment approaches and intensities [2].

The importance of physical function assessment in the practice for patients with cancer is no exception. Cancer rates are rising; 35 million are estimated to be newly diagnosed in 2024, an increase from 20 million in 2022 [3]. This trend applies not only to older people but also to younger people [4,5]. Although various guidelines suggest standard treatments according to the cancer stage, they cannot be applied uniformly because patients vary in their ability to accept invasion. Furthermore, due to global aging trends, cancer treatment decisions and clinical trial eligibility can no longer be based solely on chronological age [6]. Failure to adequately assess tolerability in patients can lead to adverse events, reduced quality of daily life after treatment, death, and decreased satisfaction among patients and their families. Moreover, inappropriate aggressive and invasive cancer treatments can cause economic losses [7]. Thus, effectively determining each patient’s ability to tolerate cancer treatment is critical; physical function is especially important.

The Eastern Cooperative Oncology Group Performance Status (ECOG PS) is the most commonly used physical function assessment tool in oncological practice [8]. ECOG PS is used to determine indications for treatment and as an eligibility criterion for clinical trials [9]. It is an easy-to-understand 5-point scale (0–4) [8] but largely depends on subjective judgment [9]. For decades, discrepancies in evaluations between those conducted by oncologists and physicians, physicians and nurses, and physicians and patients have been noted [10,11,12]. This interrater variability in ECOG PS prevents the medical team from sharing a unified treatment plan. In addition, despite the simplicity of the ECOG PS assessment, its documentation rate in electronic health records is approximately 50% for patients with colorectal cancer [13]. This percentage was markedly lower than expected.

Additionally, several measuring tools in oncological practice have been proposed for physical function assessment: the Short Physical Performance Battery (SPPB), Timed Up and Go (TUG) test, grip strength, and walking speed (4 m/6 m/8 m/10 m walking time, or 6 min walking time) [14]. These are called objective physical function assessments, which are quantitative and do not rely on the individual judgment of assessors [9,14]. SPPB is rated on a 12-point scale across three domains: 4 points for the ability to maintain closed-leg standing, semi-tandem, and tandem for 10 s; 4 points for the time to walk 4 m; and 4 points for the time required to stand and sit five times [15]. The TUG test measures the total time required to stand up from a chair, walk back and forth a distance of 3 m, and sit down [16]. Several measurement methods have been proposed for walking speed based on distance or time [14]. Grip strength was measured using a dynamometer [14].

Associations between objective physical function assessments and clinical outcomes in cancer patients have been reported. Some systematic reviews have highlighted the correlation between SPPB, TUG, walking speed, grip strength, and all-cause mortality [17,18]. In the surgical field, the SPPB is a predictor of prolonged hospital stay [19] and hospitalization-associated disability [20]. TUG is associated with severe postoperative complications and 90-day [21] or 5-year postoperative mortality [22]. In the field of chemotherapy, SPPB and grip strength predict treatment plan modifications or dose reduction [23,24,25]. The nutritional status of patients with cancer has also been associated with SPPB and grip strength [26,27]. Repeated objective physical function assessment enables the monitoring of the impact of treatment and determination of the effectiveness of rehabilitation interventions [28,29,30]. Furthermore, a decline in gait speed by 0.1 m/s results in an 8% increased risk of cardiovascular diseases [31]. Objective physical function assessment is also related to frailty and has attracted increased attention due to global aging trends [32]. Objective physical function assessment tools, such as SPPB, TUG, and walking speed, especially the 10-Meter Walk Test, are listed in the physical function domain according to the National Comprehensive Cancer Network Guidelines for Older Adult Oncology [33].

**Figure 1:**
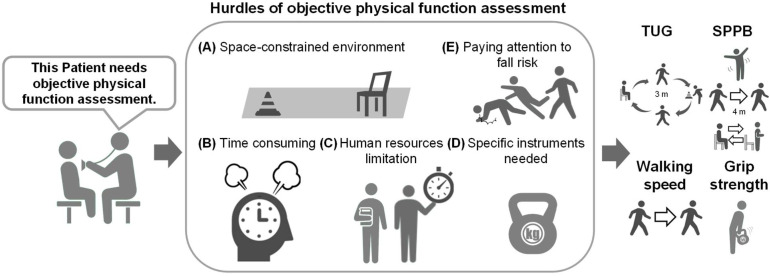
Challenges of objective physical function assessments in clinical settings. Key obstacles include: (**A**) space constraints—limited room for assessments; (**B**) time demands—assessments require significant time; (**C**) human resources—shortage of trained personnel; (**D**) specialized instruments—need for specific equipment; and (**E**) fall risk—ensuring patient safety and managing fall risk. These barriers make single assessments difficult during clinical courses. Additionally, the manual nature of these evaluations can lead to interrater variability, complicating their broader implementation.

Thus, objective physical function assessments are highly informative due to the association between their results and various clinical outcomes. Conversely, they have several limitations. Although implementing physical function assessment may seem simple, the assessor must consider the possibility of the patient falling during the evaluation. Additionally, constraints related to personnel, time, and space limit the incorporation of these assessments into hospital care, especially in outpatient settings, which hinders their implementation in daily clinical practice [1]. As mentioned earlier, changes in physical function can be monitored through repeated assessments over time [28,29,30]; however, a single assessment at one point during each clinical course may not be adequately conducted owing to these barriers (Figure 1). Consequently, objective physical function assessment tools have not been widely adopted in clinical practice. The recent COVID-19 pandemic has further strained healthcare resources, leading to a shortage of therapists, particularly those conducting objective physical function assessments [34]. Moreover, the uneven distribution of therapists across regions has resulted in healthcare disparities [35].

To address these limitations, several studies have simplified the measurement of SPPB and TUG using Doppler sensors [36], one-dimensional light detection and ranging [37], automated multiple cameras [38], and instrumented inertial measurement units [39]. While these technologies automate the process of objective physical function assessment, they do not alleviate the constraints of space, personnel, or additional equipment. Furthermore, the cameras and other tools used may not be suitable for actual clinical use in terms of functionality.

Artificial intelligence (AI) technology has garnered attention in recent years owing to advances in machine learning (ML) with the advent of deep learning; advances in information infrastructure technology, such as the emergence of inexpensive and high-performance graphics processing units; and the ease of utilizing large-scale data, such as the expansion of public databases with the advent of the big data era [40,41]. The medical field is no exception, with active medical research utilizing AI technology in various forms, including medical image analysis [42,43,44,45], omics analysis [46,47,48], and natural language processing, with a focus on electronic medical records (EHRs) [49,50]. Importantly, medical devices utilizing AI technology are being promoted in rapid succession. According to the latest U.S. FDA database, more than 800 AI-powered medical devices have been approved [51]. AI technology is also being studied for objective physical function assessment, where AI is being used to provide promising options for estimating established physical function indicators from videos of various movements, sensor data, and EHR table data without the need to perform special movements. In fact, we are also currently developing an AI model to assess a patient’s physical function from routine clinical activities, such as entering and exiting the consultation room or sitting and standing from a chair. Research on this concept is novel, making it essential to explore which types of data—whether video, images, sensor data, or clinical information—can effectively predict objective physical function using AI. Therefore, in this review, we will discuss the effectiveness and issues of using AI to evaluate physical function by comprehensively surveying related papers published to date, assuming that AI technology will be used to objectively, accurately, and easily evaluate physical function. In particular, since ML is the fundamental technology behind current AI research [52], we focus on and discuss objective physical function assessments using ML technology.

## 2. Methods

### 2.1. Search Strategy

PubMed was searched in April 2024 for relevant studies using the following search strategy: (“Short Physical Performance Battery” OR “Timed up and Go” OR “grip strength” OR “walking speed” OR “gait speed” OR “fall risk”) AND (“artificial intelligence” OR “machine learning” OR “deep learning”). The titles and abstracts of the selected papers were searched.

### 2.2. Selection Criteria

The inclusion criteria for selecting the papers were that they should have been written in English. The publication period was not restricted. Notably, we determined that, in light of patients’ quality of life and the current situation in clinical practice, there is a need to develop a system that uses ML to predict the results of objective physical function assessments from videos of patients’ movements in hospitals during routine medical examinations, without conducting additional assessments. Thus, we reviewed techniques that can be adopted in clinical practice without requiring specific conditions solely for assessing physical function. Specifically, this review included studies where SPPB, TUG, walking speed, and grip strength were predicted using ML from videos, sensor data, or clinical information from EHR without manual measurement or additional clinical resources. Conversely, we excluded studies that utilized ML solely to automate the evaluation process, such as those that performed TUG in front of a camera without a measurer or conducted TUG or SPPB at home with attached sensors. Similarly, studies that used inertial measurement units (IMUs) or Doppler technology to automate the assessment of SPPB and TUG to reduce human resources were excluded. The same applied to walking speed, where the goal was to simplify the measurement of the 6 min walk or 10 m walk. From another perspective, several studies used ML to segment successive movements (such as standing up, walking, and turning) and extract gait parameters from IMU data or videos related to SPPB, TUG, and walking speed. Additionally, studies that used grip strength as an explanatory variable to predict SPPB scores or past TUG test scores and to predict future TUG test scores were included in the review. Only recent studies by the same authors were included, while studies by the same authors with different concepts were also included. Predicting gait speed posed challenges owing to the different movements involved. Therefore, we selected studies proposing systems for future use in clinical practice.

## 3. Results

### 3.1. Overall Trends in AI-Based Objective Physical Function Assessment Research

The initial search yielded approximately 370 articles with the following distribution: SPPB, 11; TUG, 68; fall risk, 132; walking or gait speed, 116; and grip strength, 40. In most studies, SPPB, TUG, and walking speed/gait speed were considered explanatory variables for predicting clinical outcomes such as survival and complications using ML. From these results, we identified 26 articles that fit our objective physical concept without performing the assessment process itself. A summary of these results is presented in Table 1. The predicted labels and number of studies were as follows: 9 papers on TUG or SPPB, 2 papers on grip strength, and 15 papers on walking speed. Over half of these studies used sensors such as IMUs, smartphones, smart home sensors, smartwatches, global navigation satellite systems (GNSSs), and Kinect V2 as input data. Most selected studies employed internal validation methods like k-fold cross-validation (CV), leave-one-out CV, train–test split, or train–val–test split. To evaluate the classification model, metrics such as accuracy, F1-score, and area under the ROC curve (AUC) were used. Common metrics for evaluating the regression model included errors and correlation indices. We summarized the results in the following sections, focusing first on the labels and then on the input data.

### 3.2. Characteristics of Studies Setting TUG/SPPB as an Output Label

Of the studies that used ML technology to objectively evaluate physical function, nine used TUG or SPPB as the output label (Table 1) [53,54,55,56,57,58,59,60,61]. Five of these studies used sensor data as input data: IMU, smartphone as an inertial sensor, and Kinect V2 as the depth sensor. In contrast, one study used videos of walking back and forth in front of a stereo camera as input data [61]. All labeled data were measured manually. Of the eight studies that used the TUG test as the label, five used classification tasks, and three used regression tasks. However, of the two papers that used the SPPB as the label, one used regression, and the other used classification. For the classification of SPPB and TUG, the cutoff values were set independently for each study: TUG, 10/13.5/14/20 and SPPB, six (male)/nine (female). As a distinctive label setting, Bloomfield et al. performed a classification for patients undergoing total knee arthroplasty (TKA) with a TUG test preoperatively and postoperatively and determined as a label whether there was an improvement of 2.27 s [55].

The ML techniques employed in most studies include orthodox techniques, such as support vector machine and elastic net, and ensemble methods, such as AdaBoost and XGBoost. Friedrich et al. used long short-term memory (LSTM) and a convolutional neural network (CNN) with sensor data [54]. Hasegawa et al. used commercial machine learning software with no coding required, such as Prediction One version 3.0.1.3 (Sony Corporation, Tokyo, Japan) [58].

The characteristics of the dataset cohort included preoperative orthopedic patients, older patients in institutions or receiving outpatient care, patients with cancer, and patients with neurological diseases such as facioscapulohumeral muscular dystrophy. Friedrich et al. used the OTAGO dataset of older adults with frailty, which is an existing open-source dataset [54,79].

### 3.3. Characteristics of Studies Setting Grip Strength an Output Label

Two studies on grip strength used regression analyses [62,63]. Hwang et al. used variables from physical profiles and body part measurements as input data for 164 healthy young volunteers [62]. In this study, various combinations of variables, including demographic and anthropometric information and posture, were tested and compared for each participant, and an attempt was made to propose the model with the highest predictive power. The methods used were MLP regression and three different polynomial regressions, and the results of comparing the performance of the regressions showed that including all the variables performed better than other combinations of variables. In addition, MLP regression showed higher performance than polynomial regression, and MLP regression that considers all variables, achieved the best performance in grip strength prediction. Bae et al. used national big data from billions of people aged over 65 years, called the Korean National Fitness Award data [63]. The aim was to determine the best ML regression model for predicting grip strength in adults aged 65 years and over using various independent variables, such as body composition, blood pressure, and physical ability. The dependent variable was grip strength, and it was shown that the CatBoost Regressor had the lowest mean square error and the highest R^2^ value among the seven prediction models tested.

These results show that a regression model based on ML can accurately predict the grip strength of older adults and may be useful for reducing the risk of musculoskeletal disorders of the upper limbs.

### 3.4. Characteristics of Studies Setting Walking Speed an Output Label

Similarly to studies that set the SPPB/TUG as an output label, the most common types of input data were recorded by the IMU, IMU with GNSS, smartphone as an inertial sensor, smartwatch, or 3D optical motion capture. However, two studies employed images, such as plantar pressure images and silhouettes of walking individuals [75,76], and the same number of studies used videos that tracked participants’ walking as input data [77,78]. For measuring walking speed as label data, Soltani et al. used GNSS measurements [65]; Kidzinski et al. used the Vicon (OMG plc, Oxford, UK) system [77,80], an optical motion capture system; and Lonini et al. used the GAITRite system, a gait analysis system with an electronic walking mat [78]. Other studies used manual measurements or treadmill gait settings. Canonical measures of walking speed included the 25-foot walking test [64], 10 m walking test [70], and 6 min walking test [68,71]. Eleven studies predicted walking speed as a regression task, while four studies approached it as a classification task. As a distinctive label setting, Lee et al. established the cutoff point for the classification task based on the difference between the preoperative and postoperative walking speeds for patients undergoing TKA; the difference was categorized as either an increase of over 10%, a decrease of over 10%, or neither [73]. Davis et al. performed a regression using the gait speed reserve of adults aged > 50 years in Ireland, which was defined as the maximum gait speed minus the usual gait speed [74].

For studies that obtained input data from sensors, ML techniques were similar to the general types used in the SPPB/TUG studies. However, for studies using images as input data, Sikandar et al. employed Bidirectional LSTM [75], while Chen et al. utilized CNN [76]. Both studies that used videos as inputs relied on pose estimation models. Kidzinski et al. compared the accuracy of walking speed estimation with CNN, random forest (RF), and Ridge, using features extracted via OpenPose [77]. Lonini et al. implemented DeepLabCut, an open-source pose estimation model with a graphical user interface [78].

The most common diseases in the dataset cohort were neurological disorders such as stroke [66,78], multiple sclerosis (MS) [64,68,70], and cerebral palsy [77]. Approximately half of the participants were healthy. Davis et al. used the large dataset from TILDA Wave 3 [74,81], while Sikandar et al. utilized the gait dataset from the Osaka University Institute of Scientific and Industrial Research dataset A [75,82].

### 3.5. Summary of Sensor Data Acquisition

Many studies predicting SPPB, TUG, and walking speed, but not grip strength, have used sensor data, mainly IMUs, as inputs. Smartphones and smartwatches were also used as IMUs. In studies in which the GNSS and IMU are used together, the GNSS is not counted as an IMU. The number of attached IMU sensors ranged from one to a maximum of five.

In the case of two or more IMUs, the attachment points are symmetrical. Polus et al. performed the TUG test for patients undergoing total hip arthroplasty (THA) with sensors above and below the knee bilaterally 2 weeks before and 2 weeks after surgery, classifying the TUG scores (more than 14 s or not) at 6 weeks postoperatively using 55 spatiotemporal and joint-specific metrics extracted from a series of sensor data [53]. Bloomfield et al. were part of the same research group as Polus et al., and the sensor attachment sites and extracted variables were the same. By contrast, Bloomfield et al. aimed to predict postoperative TUG improvements based on preoperative TUG tests [55]. McGinnis et al. had participants walk bilaterally on a treadmill with sensors placed above and below the knees. Seven patterns were set up with combinations of sensor positions, and a linear model trained on data from 17 healthy participants was used to estimate the 6 min walk of 30 patients with MS on the ground. The best model (root mean square error = 0.12) was developed using a single sensor on the sacrum [68].

In the case of single sensors, the site of fixation was either a shoe or a pelvic area such as the waist, sacrum, or lower back. When a smartwatch is used as the sensor, it may be fixed to the wrist [72], but Juen et al. used a smartphone fixed with a belt at the L3 level to collect data [71]. Further, Zhuparris et al. used participants’ smartphones as accelerometers but instructed them to handle them as usual without any specific fixation [56].

**Figure 2:**
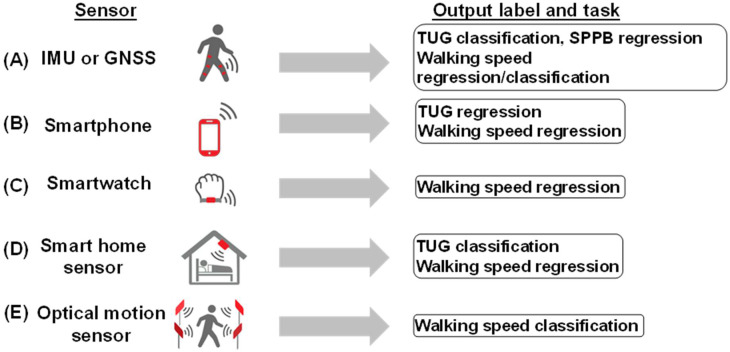
Utilization of sensor data to assess physical function. (**A**) IMU or GNSS: used for TUG classification, SPPB regression, and walking speed regression/classification. (**B**) Smartphone: employed for TUG and walking speed regression. (**C**) Smartwatch: applied for walking speed regression. (**D**) Smart home sensor: utilized for TUG classification and walking speed regression data collection in facilities and residences spanning from 8 h to several weeks for continuous monitoring. (**E**) Optical motion sensor: used for walking speed classification.

Only one study used optical sensor data as the input. Lee et al. predicted an improvement in walking speed after TKA using clinical information from the EHR and gait parameters extracted from walking at the Human Motion Analysis Lab [73]. When smart home sensors and Kinect V2 were installed in facilities and residences, the data collection period ranged from 8 h to 7 days [57,83]. In some cases, the collection lasted for several weeks (Figure 2) [84].

### 3.6. Studies with Image Input Data

**Figure 3:**
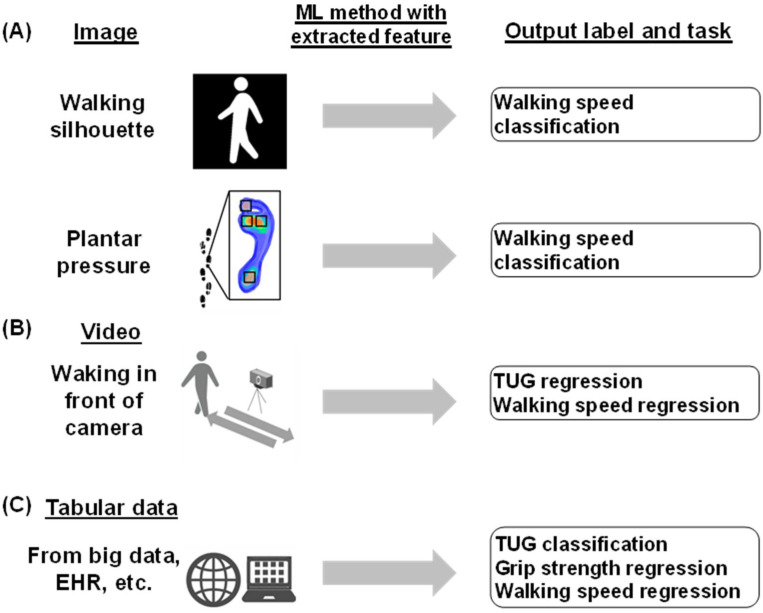
Utilization of images, videos, and tabular data in assessing physical function. (**A**) Image: walking silhouettes are used for walking speed classification. These images are from the OU-ISIR Dataset A. Plantar pressure is also used for walking speed classification. Plantar foot pressure images, including the regions of interest (T1, M1, M2, and HL), were classified using a convolutional neural network (CNN) at different walking speeds and times. (**B**) Video: walking in front of the camera, utilized for TUG and walking speed regression. Common pose estimation models, such as OpenPose and Mask R-CNN, were used for keypoint extraction. (**C**) Tabular data from big data and EHR, among others; used for TUG classification, grip strength regression, and walking speed regression.

Of the papers reviewed, two studies predicted walking speed using images as input data, whereas no studies predicting SPPB, TUG, or grip strength utilized images as input data. Sikandar et al. extracted five features (full-body height, full-body width, mid-body width, lower-body width, full-body area, apparent body area, and area between two legs) from a series of silhouette images at three different walking speeds. These five features were engineered into time series data, and a walking speed classification task was performed using bidirectional long short-term memory [75]. Chen et al. also established a region of interest for plantar foot pressure images, including the first toe, first metatarsal head, second metatarsal head, and heel. The images were classified into three levels of walking speed and two levels of walking duration using a CNN with each image as the input (Figure 3A) [76].

### 3.7. Studies with Video Input Data

Of the papers reviewed, one study predicted TUG and one study predicted walking speed using video as input data. Yuan et al. recorded facility residents walking in front of a stereo camera, extracted gait parameters using a mask R-CNN, and obtained gait speed and step length as the core features. They developed an original multiple regression model with various variables, including these core features, to estimate TUG scores [61]. Kidzinski et al. tracked and filmed the gait of 1026 pediatric patients with cerebral palsy using a single camera and obtained 1792 videos [77]. Key points were extracted using OpenPose, a common video pose estimation model, and the data for each coordinate were input as time series data to the CNN, along with summary statistics, such as the mean and percentiles for the RF/ridge regression. Gait parameters, such as walking speed and indices, were predicted. The OpenPose + CNN strategy yielded the best metrics for all prediction labels.

Lonini et al. employed DeepLabCut, a ResNet-based pose estimation model, to predict the walking speed of patients with stroke. Only the lower-body movements of the participants were recorded in the gait videos. In addition, the walking parameters measured using the GAITRite system were used as ground truth data (Figure 3B).

### 3.8. Studies with Tabular Input Data

Some studies employed tabular data as inputs. These mainly included clinical information extracted from EHRs and other sources or tabular datasets of existing datasets, including body part measurements or other objective measures of physical function as explanatory variables. Hasegawa et al. included calf circumference and grip strength as explanatory variables and conducted a binary classification task for the SPPB score with a cutoff value [58]. Kraus et al. also included the grip strength of the dominant and non-dominant hands as explanatory variables and conducted a regression analysis for the TUG score [59]. Hwang et al. conducted a grip strength regression task with variables including hand width and length in addition to age, height, and weight. Using data from the Korean National Physical Fitness Award, Bae et al. predicted grip strength using several variables, including TUG scores and walking speed (Figure 3C) [63].

## 4. Discussion

Research papers predicting the results of objective physical function assessments (TUG, SPPB, grip strength, and walking speed) using sensor data, videos, images, questionnaires, EHR items, and big data were reviewed. Most of the articles were related to walking speed and were published earlier than those related to other objective physical function assessments. Compared to SPPB, TUG, and walking speed, grip strength does not require much effort to measure if only a meter is available, which may explain why fewer studies have predicted grip strength using the ML method. However, fewer studies on the SPPB and TUG than on walking speed may be attributed to the components of the SPPB other than walking, such as maintaining balance and standing up. The TUG test includes getting up, transitioning to walking, and changing directions, making it a more challenging task to predict.

The types of objective physical function assessments and task types varied across the reviewed studies. Walking speed has been shown to correlate with disease when changes of 0.1 m/s occur [31], while grip strength changes of 5–6.5 kg may be considered clinically significant [85]. SPPB scores of 0–3 vs. 10–12, 4–6 vs. 10–12, and 7–9 vs. 10–12 are associated with differences in all-cause mortality [86]. For TUG, studies indicate that categorizing results as slow (≥15 s), intermediate (11–14 s), and fast (≤10 s) correlates with increased rates of postoperative complications and mortality temporally [87]. While it is challenging to establish a clear standard for best metrics among the reviewed studies, prediction at a finer resolution is essential.

Several studies employed IMUs and other sensors as modalities for extracting input data. Although the use of smartwatches and smartphones as sensors is reasonable, the impact of COVID-19 may present challenges in attaching things, bringing things into the living environment, and installing things touched by an unspecified number of people. In addition, smartphones have evolved remarkably in recent years; however, the challenge for older adults may still be high, and having to instruct them on the use of the application is a burden. Even if patients are instructed to wear these devices at home, the quality and quantity of the data can be unassured. However, the need for additional sensor equipment poses a barrier; using a single sensor, such as a smartphone, smartwatch, or IMU, may reduce the psychological burden on participants by minimizing the effort required for device attachment or set up.

Some studies have also used tabular data from EHRs and other sources, and these datasets include measurements of body parts or other types of objective functional assessment results (e.g., walking speed is used to predict grip strength, and conversely, grip strength is used to predict SPPB). Thus, despite efforts to resolve these challenges, other physical function assessment procedures in clinical practice may require an additional burden. In this respect, video, which is non-contact and can obtain data at a fixed point, has an advantage. However, there are challenges in clinical implementation, such as video shooting conditions that serve as inputs and the need for a video camera operator to track the walker. A video-based model that replaces the existing physical function evaluation process and results by extracting features of walking, standing, and sitting in a clinical setting would be beneficial. This would reduce the burden on medical professionals and enable more detailed observation of physical functions.

Aside from grip strength, the task of predicting physical function measures was either a classification or regression task in the reviewed studies. While classification tasks are useful for screening and determining eligibility or ineligibility for treatment by setting certain cutoff values, they fall short in expressing the degree of deviation from these cutoffs and cannot track changes in physical function over time. On the other hand, for measures like the TUG and walking speed, the amount of change—whether improvement or worsening—has been reported to correlate with clinical outcomes [88] and the risk of vascular diseases [31]. Therefore, regression tasks may be more useful than classification tasks. Even multilevel classification tasks with finer categories are preferred.

Furthermore, the distribution of the label data and the size of the dataset are very important for training ML models. Several studies have predicted walking speed using datasets from younger age groups, whereas studies predicting the SPPB/TUG have been conducted on older individuals, leading to imbalanced data. To develop a regression model that effectively screens and tracks score trends over time, careful consideration of the cohort size and participant background during research design is crucial.

Lastly, none of the reviewed studies were validated using external data. To address the limitations of the dataset size, several studies have employed k-fold CV and leave-one-out CV to evaluate model performance. Only a few studies used large datasets from self-administered centers. Most studies were based on datasets with fewer than 100 participants. In the future, it will also be important to create an environment for external validation using large-scale datasets by preparing public datasets that include tabular data, sensors, and video data related to objective physical function evaluation.

## 5. Conclusions

We reviewed the literature on the prediction of objective physical function indices such as the SPPB, TUG, grip strength, and walking speed using AI technology without performing the evaluation. If objective physical function assessment can be easily and repeatedly performed using such technologies, it will be possible not only to determine eligibility at the start of treatment but also to detect minimal changes over time, which may contribute to personalized medicine. In particular, the number of elderly cancer patients is increasing, and to determine the optimal treatment plan based on each patient’s physical function, it is crucial to develop an AI system that can accurately predict the results of objective physical function assessments from in-hospital motion videos when considering the QOL of elderly cancer patients.

## Figures and Tables

**Table 1:** Objective physical function assessment with ML technology.

Reference	Main Device to Obtain Input Data	Details of Input Variable or Device	Label Setting/Label Measurement Method	Output Label/Sort of Task	ML Technology	Validation Method	Metrics from Best Model	Baseline Characteristics	Concept	
Polus et al. [53]	IMU	4 sensors during TUG: above and below each knee before and 2 weeks after THA	TUG > 14(6 weeks after THA)	TUG/classification	LDA, SVM	10-fold CV	LDA: Accuracy 0.87	72 patients undergoing THA	Preventing falls by predicting their risk based on TUG	
Friedrich et al. [54]	IMU	Single sensor on the right side of the hip	SPPB: score itself TUG: <10, 11–19, 20–29	SPPB/regression TUG/classification	LSTM+CNN	Train–val–test	Accuracy (TUG) 95.9% Accuracy (SPPB) 94.3%	20 older patients (OTAGO study)	Predicting TUG on real-life IMU data	
Bloomfield et al. [55]	IMU+EHR	·4 sensors: above and below each knee during TUG ·Clinical information ·Patient-reported subjective measures	(Preoperative TUG—postoperative) >2.27	TUG/classification	SVM, NB, RF,	10-fold CV	RF: Accuracy 0.80	82 patients undergoing TKA	Predicting functional recovery for appropriately adjusting patient expectations	
Zhuparris et al. [56]	Smartphone	·Health-related data from smartphone ·Sensor in smartphone	TUG score itself	TUG/regression	Elastic Net, RF, xgBoost	5-fold CV	Elastic Net: R2 0.59	38 patients with FSHD	Quantifying FSHD progression with TUG	
Dubois et al. [57]	Depth sensor	Kinect V2 placed in each room of the rehabilitation center	TUG ≥ 13.5 s	TUG/classification	AdaBoost, NB, KNN, SVM, RF, NN	Leave-one-out CV	KNN, NN: Accuracy 1.0	30 older patients in a rehabilitation center	Preventing fall with home-sensor data	
Hasegawa et al. [58]	EHR	·Clinical information mainly from EHR ·Physical measurements	SPPB ≤ 6(men)/≤9(women) as fall risk	SPPB/classification	Prediction One. Ver3.0.1.3 (SONY) BLRA	Train–test split	Prediction One: Accuracy 0.74	797 older patients at frailty outpatient service	Comparing model performance of predicting fall risk based on SPPB	
Kraus et al. [59]	EHR	Clinical information from HER	TUG score itself	TUG/regression	GLM, SVM, RF, xgBoost	5-fold CV	RF: MAE 2.7	103 orthogeriatric patients	Predicting TUG without mobility data	
Sasani et al. [60]	Tabular data	Components of GA	TUG < 10 s, TUG ≥ 10 s, uncertain	TUG/classification	Decision Tree Classifier	None	Decision Tree Classifier: Accuracy 78%	1901 old patients undergoing cancer surgery	Predicting accurately TUG score with ML	
Li et al. [61]	Video	Stereo camera	TUG score itself	TUG/regression	Mask R-CNN+ polynomial regression	None	RE <0.1 (20 participants in 40)	40 older adults in a daycare facility	Assessing the health status of the older patients with TUG	
Hwang et al. [62]	Tabular data	Variables from physical profile and body part measurements (not from EHR)	Grip strength score itself	Grip strength/regression	MLP regression and different polynomial regressions	K-fold CV	MLP regression: correlation 0.88	164 healthy young volunteers	Predicting grip strength accurately to reduce the risk of upper extremity disorder	
Bae et al. [63]	Big Data	Tabular data from Korean National Fitness Award Data from 2009 to 2019	Grip strength score itself	Grip strength/regression	LR, LASSO, Ridge, RF, xGBoost, Light GBM, CatBoost	5-fold CV	CatBoost: MSE 16.6	107,290 participants aged over 65	Predicting grip strength without measuring	
Supratak et al. [64]	IMU	Single sensor on the lower back	25-foot walking test in clinic	Walking speed/ regression	SVR	Correlation	Correlation 0.98	32 young patients with MS	Validating gait speed at home against a 25-foot walking test	
Soltani et al. [65]	IMU+GNSS	2 sensors: on each wrist	Walking speed measured by GNSS	Walking speed/ regression	LASSO (feature extraction)	CV	RMSE 0.05	40 healthy young volunteers	Estimating walking speed with personalization	
Dobkin et al. [66]	IMU	2 sensors: above each ankle	Walking speed measured by stopwatch	Walking speed/ regression	Sensor system (Medical Daily Activity Wireless Network algorithm)	Correlation	Correlation 0.98	12 patients with stroke 6 healthy participants	Acquiring quantitative data on daily performance	
Mannini et al. [67]	IMU	Single sensor on the right shoe	Walking speed manually measured	Walking speed/ regression	·Hidden Markov model ·Strap-down integration ·LR	Leave-one-out CV	R2 0.96	23 healthy adults	Exploring the ML method to predict walking speed	
McGinnis et al. [68]	IMU	5 sensors: on sacrum, bilateral thigh, and bilateral shank	6 min walking test on a treadmill	Walking speed/ regression	SVR	Leave-one-out CV	RMSE 0.12 (patients with MS)	17 healthy participants 30 patients with MS	Resolving the hurdle of assessing walking speed	
Aziz et al. [69]	IMU	Single sensor inside one shoe	Slow/normal/fast speed	Walking speed/ classification	RF, xgBoost, SVM	Train–test split	RF: Accuracy 1.0	10 healthy men	Analyzing gait patterns of aged people	
Atrsaei et al. [70]	IMU	Single sensor on the waist	10 m walk test	Walking speed/ regression	GPR	Leave-one-out CV	RMSE 1.10	35 participants with MS	Predicting walking speed at home with IMU	
Juen et al. [71]	Smartphone	Smartphone in waist belt at L3	6 min walking test	Walking speed/ regression	SVM, GPR	Leave-one-out CV	SVM: Error 3.23	28 patients with pulmonary disease 10 healthy participants	Monitoring individual health status continuously	
Aziz et al. [72]	Smartwatch	Smartwatch on the right wrist	Speed during treadmill walking: 0.5, 0.75, 1.0, 1.25, 1.5, 1.75 m/s	Walking speed/ regression	GPR	None	MAPE 4% (best, 1.0 m/s)	10 healthy young adults	Assessing walking speed for preventing chronic diseases	
Lee et al. [73]	Optical motion capture+ EHR	·Clinical information from EHR ·Variables extracted from optical motion capture	The difference between post/pre-operative gait speed	Walking speed/ classification	GBM	10-fold CV	AUC 0.86	128 female patients undergoing bilateral TKA	Predicting postoperative walking speed by preoperative clinical variable	
Davis et al. [74]	Big Data	Tabular data	GSR = MGS—UGS	Walking speed/ regression	HGBR	5-fold CV	R2 0.21	3925 participants from TILDA wave3	Predicting gait speed from population statistical data	
Sikandar et al. [75]	Image	5 ratio-based body measurement from marker free video images	Slow (2 to 3 km/h), normal (4 to 5 km/h), and fast (6 to 7 km/h)	Walking speed/ classification	BiLSTM	17-fold CV	Accuracy 92.79%	34 participants (OU-ISIR dataset A)	Classifying walking speed with body measurements	
Chen et al. [76]	Image	Plantar region pressure images	(0.8, 1.6, 2.4 m/s) and (10, 20 min)	Walking speed/ classification	ROI+CNN	Train–test split	F1-score: 1.00 (first toe, 2.4 m/s for 10 min)	12 healthy young participants	Detecting appropriate exercise intensity	
Kidzinski et al. [77]	Video	Timeline keypoint data derived from OpenPose	Walking speed measured by the VICON system	Walking speed/ regression	OpenPose+ (CNN/RF/Ridge)	Train–val–test	OpenPose+CNN: Correlation 0.73	1026 pediatric patients with cerebral palsy	Simplifying the quantitative gait assessment	
Lonini et al. [78]	Video	Below-waist videos of patients recorded by normal camera	Walking speed measured by GAITRite	Walking speed/ regression	DeepLabCut(ResNet based)	Leave-one-out CV	Correlation 0.92	eight patients with stroke	Predicting the walking speed of patients with stroke without expensive instrument	

Abbreviations: ML, machine learning; IMU, inertial measurement unit; EHR, electronic health record: GA, geriatric assessment; TUG, Timed Up and Go test; SPPB, Short Physical Performance Battery; LDA, linear discriminant analysis classifier; SVM, support vector machine; LSTM, long short-term memory; BiLSTM, bidirectional long short-term memory; CNN, convolutional neural network; NB, naive Bayes classifier; RF, random forest; xgBoost, eXtreme gradient boosting; AdaBoost, adaptive boosting; KNN, k-nearest neighbors; NN, neural network; BLRA, binomial logistic regression analysis; GLM, generalized linear model; MLP, multilayer perceptron regression; LR, linear regression; GBM, gradient boosting machine; CatBoost, categorical boosting; SVR, support vector regression; GPR, gaussian process regression; HGBR, histogram gradient boosting regression; CV, cross validation; ROI, region of interest; MAE, mean absolute error; MSE, mean squared error; RMSE, root mean square error; RE, relative error; MAPE, mean absolute percentage error; AUC, area under the curve; R2, R-squared value; ICC, intraclass correlation coefficient; GSR, gait speed reserve; MGS, maximum gait speed; UGS, usual gait speed; GNSS, global navigation satellite systems; TKA, total knee arthroplasty; THA, total hip arthroplasty; FSHD, facioscapulohumeral muscular dystrophy; MS, multiple sclerosis; OU-ISIR, Osaka University Institute of Scientific and Industrial Research; TILDA, The Irish Longitudinal Study on Aging.

## Data Availability

No new data were generated for this study.
